# Editorial: Advances in robotic surgery: innovations, applications, and future directions

**DOI:** 10.3389/fsurg.2025.1740369

**Published:** 2025-11-20

**Authors:** Basilio Pecorino, Antonino Maniaci, Annalisa Umile, Luigi La Via, Antonio Zanghì

**Affiliations:** 1 Department of Medicine and Surgery, Kore University of Enna, Enna, Italy; 2 Department ChirMED, Gaspare Rodolico Hospital, University of Catania, Catania, Italy; 3 Department of Medical, Surgical Sciences and Advanced Technologies, University of Catania, Catania, Italy

**Keywords:** robotic surgery, anesthesiology, artificial intelligence, perioperative management, intelligent operating room, surgeon’s performance

## Abstract

Robotic surgery has transformed modern operative practice by enhancing precision, dexterity, and visualization far beyond traditional surgical methods. This article explores the evolving integration between robotic surgery and anesthesiology, emphasizing the multidisciplinary nature of perioperative management in technologically advanced environments. As robotic platforms become increasingly intelligent—incorporating artificial intelligence, real-time data feedback, and automation—anaesthetic practice must adapt to new physiological, logistical, and ethical challenges. The development of smart operating rooms and closed-loop anesthesia systems highlights the growing interdependence between surgical and anesthetic processes. Moreover, simulation-based education and interprofessional teamwork are essential to mastering the robotic learning curve and ensuring patient safety. Economic sustainability and ethical governance remain critical as automation and AI reshape surgical responsibility and patient consent. Ultimately, the convergence of robotic systems, anaesthetic innovation, and artificial intelligence defines the future of intelligent perioperative integration, where human expertise and adaptive machine precision cooperate to achieve safer, more efficient, and personalized surgical care.

## 1 Introduction

Robotic surgery has revolutionized modern operative practice, offering unmatched precision, dexterity, and visualization beyond the limits of traditional techniques. This research topic, “Advances in Robotic Surgery: Innovations, Applications, and Future Directions,” brings together multidisciplinary contributions that explore the evolution, challenges, and future trajectories of robotic-assisted surgery, with growing attention to anesthesiologic integration. (1) As robotic technology becomes increasingly sophisticated, incorporating artificial intelligence (AI), haptic feedback, and enhanced visualization, it not only transforms the surgeon's performance but also reshapes anaesthetic and perioperative management (2). Robotic surgery is inherently multidisciplinary, and its success depends on the seamless collaboration between surgeons and anesthesiologists.

## 2 Expanding surgical and anesthesiologic frontiers

Originally pioneered in urology and gynaecology, robotic surgery has expanded across general, cardiothoracic, head and neck, and orthopedic specialities. Each of these fields presents unique physiological and logistical challenges that directly affect anaesthetic practice. Robotic procedures often require steep Trendelenburg positioning, pneumoperitoneum, prolonged operating times, and limited patient access once the robot is docked, all of which pose specific anaesthetic implications Lao et al. Hemodynamic stability, airway management, and ventilation under high intra-abdominal pressure must be carefully maintained. In head and neck and thoracic robotic approaches, anesthesiologists play a crucial role in ensuring airway safety, rapid communication during docking and undocking, and readiness for emergency interventions. Thus, anesthesiology is not merely supportive but integral to the robotic workflow, contributing actively to patient safety, surgical precision, and procedural efficiency.

## 3 Technological integration and the smart operating room

Technological advancements in robotic systems, such as 3D optics, motion scaling, and tremor filtration, are paralleled by progress in anaesthetic monitoring and automation Takahashi et al. Modern anaesthesia workstations increasingly integrate physiologic data with surgical navigation systems, enabling continuous feedback loops between anaesthetic depth, ventilation parameters, and surgical manipulation. Several contributions within this Topic explore the emerging role of AI, machine learning, and computer vision in perioperative management. Predictive algorithms can forecast hemodynamic instability, optimise anaesthetic drug delivery, and adjust ventilatory settings in real time. These capabilities are paving the way for the smart operating room, in which surgical and anaesthetic systems communicate synergistically to enhance safety and precision.

## 4 Training, learning curve and human factors

The robotic learning curve encompasses both the surgical and anesthetic teams. Transitioning to robotic platforms requires anesthesiologists to master new positioning, workspace limitations, and emergency protocols. Simulation-based training, virtual reality, and team-based crisis resource management exercises are now essential components of robotic education Xiao et al. This Topic's contributions emphasize that robotic proficiency is fundamentally a team skill. Multidisciplinary curricula must foster situational awareness, interprofessional communication, and shared decision-making in technologically complex environments. Only through such cooperative learning can surgical and anesthetic teams achieve the precision and coordination demanded by robotic systems.

## 5 Economic and organizational considerations

The widespread adoption of robotic surgery carries significant economic implications. Although acquisition and maintenance costs remain high, several studies within this Topic note that cost-effectiveness analyses should also account for reduced postoperative complications, shorter hospital stays, and optimized anesthetic recovery Huang et al. From an organizational standpoint, anesthesiology departments are pivotal in improving robotic workflow efficiency. Rapid induction and emergence protocols, optimized turnover times, and standardized perioperative pathways directly influence institutional sustainability and patient throughput. As robotic systems become increasingly modular and mobile, anesthesiologists will continue to shape operating room logistics and safety frameworks.

## 6 Ethical, legal and human dimensions

The integration of AI and automation into surgical and anesthetic systems introduces profound ethical and legal challenges. Questions regarding liability in the event of AI-driven errors, data privacy, and informed consent are increasingly complex. While automation can improve efficiency and reduce human error, preserving clinician oversight and empathy remains essential. Emerging concepts such as tele-anesthesia and remote intraoperative monitoring require rigorous validation and ethical governance to ensure patient safety. As robotic and anesthetic technologies evolve, maintaining transparency, accountability, and patient-centeredness will be key to responsible innovation.

## 7 Future directions: towards intelligent perioperative integration

The convergence of surgical robotics, anesthetic automation, and artificial intelligence heralds the next era of operative care. Research is now exploring semi-autonomous robotic actions, closed-loop anesthetic delivery, and AI-assisted airway management (3). These developments promise to enhance safety, extend access to expert care, and standardize complex procedures even in remote or resource-limited settings. Achieving this vision will demand strong collaboration among engineers, surgeons, and anesthesiologists to define interoperability standards, validate algorithms, and ensure clinical accountability. The operating room of the near future will likely function as an intelligent ecosystem, combining human expertise with adaptive machine precision.

## 8 Conclusion

The articles in this research topic collectively demonstrate that the progress of robotic surgery is inseparable from anesthesiologic innovation. Together, these disciplines form a model of cooperative intelligence in the operating room, where human skill, clinical insight, and technology merge to achieve outcomes once considered impossible. As surgical and anesthetic teams continue to innovate, train, and collaborate, robotic surgery will advance toward a future of safer, smarter, and more personalized patient care.
